# Impact of ISO/IEC 17025 laboratory accreditation in sub-Saharan Africa: a case study

**DOI:** 10.1186/s12913-020-05934-8

**Published:** 2020-11-23

**Authors:** Mercy A. Okezue, Mojisola C. Adeyeye, Steve J. Byrn, Victor O. Abiola, Kari L. Clase

**Affiliations:** 1 School of Agricultural & Biological Engineering, Biotechnology Innovation & Regulatory Science, Purdue University, West Lafayette, USA; 2 Bindley Bioscience Centre, Purdue University, 1203 W State St, West Lafayette, IN 47907 USA; 3 National Agency for Food and Drug Adm. & Control, NAFDAC, Abuja, Nigeria; 4 Department of Industrial & Physical Pharmacy, Purdue University, West Lafayette, USA

**Keywords:** Accreditation, ISO/IEC17025: 2005, Audits, Quality control laboratory, Nonconformities, Sub-Saharan Africa

## Abstract

**Background:**

The number and severity of nonconformities generated during an audit of a medicine testing laboratory indicates its level of quality compliance. Quality standards are established and maintained to ensure the reliability of laboratory test reports. The National Medicines Regulatory Authority (NMRA) Quality Control laboratories assess the quality of medicines used by the populace as part of their regulatory function. Although countries desire to have reliable medicine testing facilities, accrediting a national laboratory to international standards poses financial and technical challenges for many low-income countries**.** Sharing the benefits of laboratory accreditation could help more countries within sub-Saharan Africa overcome existing challenges to achieve accreditation and robust quality systems. This study investigated the impact of ISO/IEC 17025 accreditation on the performance of an NMRA Quality Control laboratory to provide evidence of improved quality compliance within a low-resource setting.

**Methods:**

Pre- and post- accreditation audits of nonconformities for management and technical requirements of the ISO/IEC17025:2005 standards were evaluated from a Quality Control laboratory in the National Agency for Food and Drug Administration and Control (NAFDAC), located in Nigeria, West Africa. The following research questions were addressed: “does accreditation impact the adherence to quality standards?” and “does accreditation decrease the severity of nonconformities in Quality Control laboratory audits?”

**Results:**

Statistical analysis of the pre- to post- accreditation audits from the years 2013 through 2017 revealed a significant decrease in the total number of nonconformities (χ^2^ = 74, *p*-value = 9.99e-05, *r* = 0.67). Further examination of audits from the years 2013 through 2018 audits also revealed a reduction in the number of nonconformities (χ^2^ = 53, *p*-value = 9.99e-05, *r* = 0.62). A reduction in the number of major observations and a decrease in the severity of nonconformities was also observed.

**Conclusions:**

A higher level of quality compliance was exhibited for the laboratory during the post-accreditation years. Overall, ISO/IEC 17025 accreditation of the NMRA Quality Control laboratory resulted in improved reliability of test reports and enhancement of the laboratory quality system.

**Supplementary Information:**

The online version contains supplementary material available at 10.1186/s12913-020-05934-8.

## Background

Institutions that conduct medicines testing apply international standards to improve the processes in their Quality Control laboratories. Laboratory accreditation can help ensure the quality of analytical reports generated by such testing facilities. In West Africa, like many parts of the world, the Quality Control laboratories of the National Medicines Regulatory Authorities (NMRA) have the task of verifying the quality and purity of medicines. Government and private institutions submit pharmaceutical products for testing to the national laboratories for regulatory compliance and other purposes. Accreditation of NMRA Quality Control laboratories provides credibility to the results generated by these government agencies and helps build practices that support the reliability of data across multiple accredited laboratories [1].

To attain this international recognition, laboratories develop their quality procedures using standards such as ISO/IEC 17025 and guidance from the World Health Organization (WHO), namely ‘Good Practices for Pharmaceutical Quality Control Laboratories’ (GPPQCL) [2–8]. The ISO/IEC 17025 standard provides requirements for laboratories to demonstrate competency and the ability to generate valid results [6].

Testing laboratories develop and maintain their systems through accreditation to international quality standards. A laboratory’s accreditation confirms its competence to carry out tests or calibrations aligned with recognized standards [4]. Establishing and maintaining these quality management systems are beneficial as these organizations can demonstrate traceability of their measurements to international standards. One of the purposes of establishing these standards is to ensure the accuracy of test results [9]. Several studies have discussed the importance of accreditation as a tool to improve the management systems in the laboratories that conduct tests and generate reports on the quality of medicines used for maintaining public health [3, 5, 10–15].

In a similar manner, educational and public health institutions that offer testing facilities also seek national and international recognition for competence in services they provide by attaining accreditation. The following reports from higher education institutions (HEI) provide an evaluation of the HEI levels of preparedness to attain ISO/IEC 17025 accreditation. Putri and colleagues, from Sebelas Maret University in Indonesia, conducted a gap analysis for the institution’s testing laboratories [16]. Similarly, Zapata-García and colleagues from the University of Barcelona, used requirements of the ISO/IEC 17025 standard to identify areas of deficiencies in their laboratory processes [17]. Areas within these institutions’ framework were identified that required training and implementing ISO standard for improved efficiency and reliability of the HEI testing laboratories [16]. Both institutions had similar objectives with aspects of this study and using laboratory accreditation as a tool to improve accuracy and mutual recognition of test results.

In further efforts that endorsed accuracy of test reports through accreditation or certification, several researchers examined laboratories handling biological samples. The Korean certification body reported that the four-year variance index score was significantly different between laboratories with or without accreditation [18]. Their results were similar to research that associated laboratory standardization with accuracy of test results [18–20]. Researchers in other studies investigated food testing laboratories and found similar outcomes [21–24].

After review of several studies, however, a paucity of literature related to the medicines’ regulatory space was observed. This region of the world encounters several barriers and cultural challenges in implementing international standards [25]. Burdick reported similar challenges in educating health professionals in Africa, noting both an unstable supply of electricity and an inadequate information system [26]. Despite these difficulties, some countries within the sub-region have attained milestones through laboratory accreditation. In Nairobi Kenya, Kibet and colleagues reported improvements in four performance parameters measured within a few years of attaining hospital laboratory accreditation [27]. Similarly, a group from the Stepwise Laboratory (Quality) Improvement Process Towards Accreditation (SLIPTA) program, highlighted a 55% compliance rate in 70% of laboratories audited by the World Health Organization Regional Office for Africa (WHO AFRO) [28]. Some low-income countries adapt the WHO AFRO SLIPTA program as a tool for implementing quality management systems [29, 30].

Despite the studies noted above, there are limited reports on the performance of Quality Control laboratories in sub-Saharan Africa. The purpose of this study, therefore, was to examine empirical data and evaluate the impact of ISO/IEC 17025 accreditation on a Central Drug Control Laboratory (CDCL) of the National Agency for Food and Drug Administration and Control (NAFDAC), Nigeria’s NMRA.

## Method

### Study objectives

1. Show that the accreditation process influenced laboratory performance.

2. Determine if some types of nonconformities (major and minor) improved more, or less than others.

3. Determine if there was evidence of a significant post-accreditation change in the number (#) of nonconformities (2017 vs. 2018).

### Research design

The present study was descriptive and comparative in design. It evaluated pre- and post- accreditation audit nonconformities (NCs) for management and technical requirements of the ISO/IEC 17025:2005 Standards. Due to the limitation of publicly available data, a convenience sampling method was used to select an NMRA Quality Control laboratory in West Africa with ISO/IEC 17025 Accreditation. Data in audit reports from a pre-accreditation year (2013), were compared to two post-accreditations years (2017 and 2018). Parametric methods were used to compare the numbers and types of non-conformances obtained from the laboratory audit reports reviewed. Certified true copies of the audit reports were provided by the Quality Assurance unit of the laboratory. The goals, hypothesis, and research questions for the study were:

### Hypothesis

ISO/IEC 17025:2005 Accreditation improves the quality management systems in a NMRA Quality Control laboratory.

### Research questions

1. Does accreditation of a Quality Control laboratory lead to improvement in the adherence to quality standard (ISO/IEC 17025:2005).
  H_0_: number (#) of nonconformities post-accreditation audits = number (#) of nonconformities pre-accreditation audits
  H_A_: # of nonconformities post-accreditation audits < # of nonconformities pre-accreditation audits
2. Does accreditation decrease the severity of nonconformities, as defined by major/minor observations or citations, in quality control laboratory audits?
  H_0_: # of major/minor nonconformities (post-accreditation audit) = # of major/minor nonconformities (pre-accreditation audit)
  H_A_: # of major/minor nonconformities (post-accreditation audit) < # of major/minor nonconformities (pre-accreditation audit)

### Object of conformity assessment [participant]

In preparing the NAFDAC CDCL for accreditation, the United States Pharmacopeia Promoting the Quality of Medicines team (USP PQM) used the 2005 standard to assess the status of the laboratory in 2013 [31]. Thereafter, the NMRA laboratory obtained ISO/IEC 17025:2005 Accreditation in 2015 and had surveillance and external mock audits performed in 2017 and 2018, respectively. The 2013 and 2018 audit were conducted by USP PQM as an external body assessment that compared the laboratory’s performance against requirements of the ISO/IEC 17025:2005 standard. A third-party surveillance audit was also performed by ANSI National Accreditation Board (ANAB) in 2017 [32, 33].

The laboratory was accredited in eleven test scope areas in 2015, with an increase to sixteen areas of scope in 2018. Details of the test scope areas can be found at the International Laboratory Accreditation Cooperation (ILAC) website [4].

### Measure

The instrument used for the laboratory assessment was the ISO/IEC17025:2005 Standard [6]. The document contains 25 clauses or audit items divided into two main parts: Management [4.0] and Technical [5.0] requirements.

Recently, many ISO standards were reviewed to align with the requirements of the ISO 9001 ‘parent’ document and a current version of the ISO/IEC 17025 standard was published to replace the older 2005 version. Several authors [34–37] have described the current 2017 version [38] as risk-focused. The 2017 version replaced the older 2005 document [6] containing two major components: management and technical requirements. The audit reports reviewed in this study were generated when the 2005 version was applicable.

For ISO/IEC 17025:2005, all activities related to legal, financial and other administrative responsibilities in running a laboratory are contained in the management requirements, while those tasks that affect how tests are conducted are contained in the technical requirements. Audits are commonly used to assess the compliance level of a laboratory and include the identification of nonconformities when the laboratory fails to meet standard requirements [39–41]. Nonconformities are classified into three categories [major, minor or opportunities for improvement (OFI)], depending on the severity of their impact on the quality of laboratory results [42]. The nonconformities that have the greatest potential to negatively impact the quality of laboratory testing are termed major. Minor nonconformities have lower risk outcomes, while other observations are opportunities for improvement (OFIs) [43].

Unlike an accreditation audit, post- accreditation assessments might not include a detailed check for each item in the 25 clauses of ISO/IEC 17025. This risk-based approach for most post-accreditation audits is based on an evaluation of the internal audit reports from the laboratory quality system. The high-risk areas identified from the internal audit reports provides areas of focus for the current audit.

### Procedure

Laboratory audit reports from assessments performed by ISO/IEC 17025 certified auditors were evaluated and secondary data analysis was conducted on one pre-accreditation report from 2013 and two post accreditation reports from the years 2017 and 2018. At the time of this study, though CDCL has undergone other audits, the three reports used for the analysis were the only ISO 17025:2005 standard assessments they had undertaken. All reports were obtained from the Quality Assurance unit of the NMRA. The laboratory audit reports were evaluated by grouping the identified nonconformities into the corresponding ISO/IEC 17025 clauses for management [4.1–4.15] and technical [5.1–5.10] requirements. The nonconformities were also categorized as major, minor and OFI (opportunity for improvement) as documented in the audit reports. The number of pre- and post-accreditation nonconformities recorded in the audit reports were analyzed. Effect sizes were used to compare the direction and relative magnitude that the independent variable (ISO/IEC 17025 accreditation, evaluated via audits) had on the dependent variable (pre- and post-accreditation accreditation number of nonconformities).

### Data analysis

The numbers and severity of nonconformities in each year audit report were counted manually, and tables of the total counts were created. The following assumptions were made: each year’s findings were drawn from a normally distributed population of audit observations and the number of nonconformities in the three audit reports have a common variance.

Analysis of Variance (ANOVA) was used to test the null hypothesis that all group means are the same, or the number of nonconformities were the same for the pre- and post-accreditation years at 95% confidence interval, α = 0.05. A Chi-square contingency test with simulation-based *p*-value, robust to small cell-counts, compared the total nonconformities between pre- to post-accreditation years. All statistics in our study were computed using Microsoft Excel 2010 edition, R studio [44] and effect size were obtained using the UCCS calculator [45].

## Results

### Research question one

Does accreditation of a Quality Control laboratory lead to improvement in the adherence to a quality standard (ISO/IEC 17025:2005)?

ISO/IEC 17025:2005 standard has 25 clauses; activities related to legal, financial and other administrative obligations in running a laboratory are contained in the management requirements while tasks related to testing activities are contained in the technical requirements. Table 1 summarizes the total nonconformities assessed in this study. Management and technical ISO clauses where nonconformities were observed from the 2013 pre-accreditation audit included both data integrity issues and inadequate personnel and training records. The specific nonconformities identified are outlined in Tables S1, S2, and S3 (see Additional file 1). Many of these operational errors were not part of subsequent post-accreditation citations from the 2017 surveillance and 2018 mock audits, as illustrated in Tables S4, S5, S6, S7, and S8 (see Additional file 1).

**Table 1:** Summary Statistics

	Year		
	2013	2017	2018
ISO clauses audited (#)	25	25	25
Total NCs	93	7	14
Mean # NCs	3.72	0.28	0.56
Std. Dev.	2.65	0.54	0.92

Summary of nonconformities for one pre- and two post-accreditation audits. Twenty-five (25) ISO/IEC 17025 clauses were evaluated each year. The total # of nonconformities decreased post accreditation years, 2017 and 2018

As shown in Table 2, the total number of nonconformities documented in the audit reports for management (mgt.) and technical (tech.) requirements were: 2013 (mgt. = 46, tech. = 47), 2017 (mgt. = 3, tech. = 4) and 2018 (mgt. = 6, tech. = 8). Analysis of variance for the pre- to post-accreditation data, Table 3, yielded a significant *p*-value (one-way ANOVA: F_2,3_ = 1140, *p* = 4.76e-05, R^2^ = 0.9987). Moreover, the high coefficient of determination, R^2^, indicated that the reduced total number of citations was attributable to the difference between the pre- and post-accreditation audits.

**Table 2:** Summary of management and technical nonconformities

# of NCs	2013	2017	2018
Management Requirements	46	3	6
Technical Requirements	47	4	8
Total # of NCs	93	7	14

Total number of management and technical nonconformities (NCs) for the laboratory improved pre- to post-accreditation

**Table 3:** ANOVA summary

Source of Variation	SS	df	MS	F	*p*-value	R^2^
Between Years	2281	2	1140	1140	4.76e-05*	0.9987
Within Years	3	3	1			
Total	2284	5				

*significant *p*-value for comparing nonconformities between audit periods

A Chi-square contingency test with simulation-based *p*-value, robust to small cell-counts, was used to compare the total nonconformities between pre- to post-accreditation years and determine which audit years had significant changes. Total nonconformities between ‘2013 vs 2017’, ‘2013 vs. 2018’ and ‘2017 vs. 2018’ audit reports were compared. As shown in Table 4, the results of these comparisons revealed that the decrease in the number of nonconformities from the pre-accreditation year 2013 to post-accreditation year 2017 was significant (χ^2^ = 73.96, *p* = 9.99e-05, α = 0.05); similarly, the decrease from 2013 to 2018 was significant (χ^2^ = 58.33, *p* = 9.99e-05, α = 0.05). There was no significant difference, however, in the total number of nonconformities post-accreditation from 2017 to 2018 (χ^2^ = 2.33, *p* = 0.198, α = 0.05).

**Table 4:** Summary Chi-squared test for paired years

# of NCs	χ^2^	*p* (2-tailed)	Cohen’s *d*	effect-size *r*
Pair 1: 2013–2017	73.96	9.99e-05*	1.796	0.668
Pair 2: 2013–2018	58.33	9.99e-05*	1.592	0.623
Pair 3: 2017–2018	2.33	0.198		

Chi-squared test with simulation, compared the number of nonconformities (NCs) pre-post accreditation, gave significant *p*-values when pre-accreditation year (2013) was paired with post-accreditation years, 2017 and 2018 respectively. Non-significant *p*-value was obtained for post accreditation years comparison

*p is significant < 0.05, NB: Bonferroni corrected significance level for alpha = 0.5 is 0.05/3 = 0.017

**Fig. 1:**
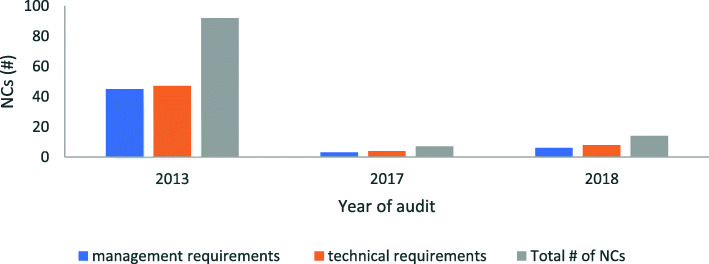
Summary of total number of nonconformities (NCs) pre- and post-accreditation. Total number of NCs for the laboratory declined pre- to post-accreditation

**Fig. 2:**
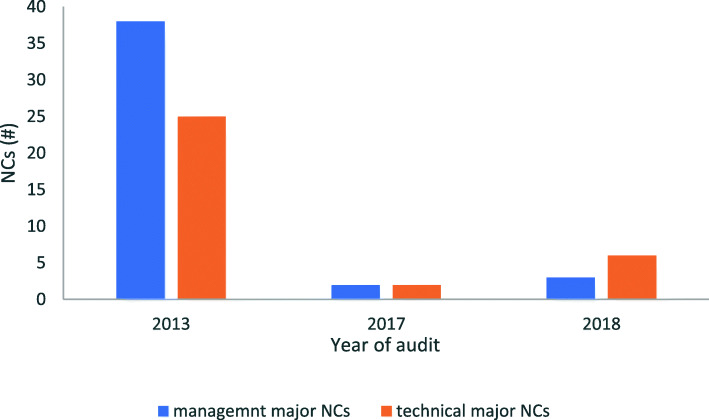
Summary for severity of laboratory’s nonconformities (NCs) pre- and post-accreditation, show major management and technical NCs decreased post accreditation. There were decreased numbers of major NCs, for both management and technical requirements, pre to post-accreditation years of audits

Following the observed decrease in nonconformities (Figs. 1 & 2) and significant *p*-values (Table 4), effect sizes were calculated for paired years: ‘2013–2017’ and ‘2013–2018’.The decrease in number of nonconformities from pre- accreditation 2013 audit to post-accreditation 2017, had a large size effect (Cohen’s d = 1.79, *r* = 0.67), similar to the 2018 audit (Cohen’s d = 1.59, *r* = 0.62) as shown in Table 4.

Following the significant *p*-values obtained for both scenarios, there was evidence to reject the null hypothesis that there was no difference in the number of nonconformities pre and post- accreditation. Therefore, the alternative hypothesis was retained since the numbers of nonconformities were lower than in the pre-accreditation era. Furthermore, the slight increase in the number of nonconformities in the post-accreditation years’ audit from 2017 to 2018, see Table 4, was not significant (χ^2^ = 2.33, *p* = 0.198, α = 0.05) and suggests the laboratory had similar citations in the post accreditation years.

### Research Question Two

Does accreditation decrease the severity of nonconformities in Quality Control laboratory audits?

In considerations for severity, the citations that have the greatest potential to negatively impact the quality of laboratory testing are termed major. Minor nonconformities have lower risk outcomes, while other observations are termed opportunity for improvements (OFI). The Tables S10, S11, S12, and S13 (see Additional file 1) detail the severity of nonconformities assessed in the laboratory audit reports.

For both management and technical requirements, as shown in Fig. 2, there were decreased numbers of major nonconformities (NCs) from pre-accreditation audit (total major NCs = 63) to the post-accreditation audit of year 2017 (total major NCs = 4), and year 2018 (total major NCs = 9), respectively. The decrease of nonconformities was significant (χ^2^ = 84.5, *p* = 9.99e-05, α = 0.05) as shown in Table 5. Therefore, there was sufficient evidence to reject the second null hypothesis that there were no changes in severity of nonconformities post accreditation. In contrast, accreditation reduced the severity of nonconformities in the laboratory. The observed increased number of major nonconformities from 2017 (NC = 4) through 2018 (NC = 9), was not significant (*p* = 0.2722, α = 0.05), which suggests that the mean number of nonconformities post-accreditation years were the same (Table 5).

**Table 5:** Summary Chi-squared test for severity of nonconformities

Total # of Major NCs	χ^2^	*p*-value
Pair 1: 2013–2017-2018	84.5	9.99e-05*
Pair 2: 2013–2017	51.96	9.99e-05*
Pair 3: 2013–2018	40.5	9.99e-05*
Pair 4: 2017–2018	1.92	0.2722

Chi-squared test with simulation compared the number of major nonconformities pre-post accreditation, and gave significant *p*-values when pre-accreditation year (2013) was paired with post-accreditation years 2017 and 2018, respectively. A non-significant *p*-value was obtained for the major NCs post-accreditation year comparison (2017–2018)

*p is significant < 0.05, *Bonferroni corrected significance level for alpha = 0.5 is 0.05/3 = 0.0167

The Additional file 1 section of this paper includes other statistical evaluations from the study, including a break-down of the management and technical requirement nonconformities, as well as their associated levels (severity) of impact on quality (see Additional file 1: Tables S6, S7, S8, S9, S10, S11, S12, and S13). Additionally, the changes in the number and severity of the laboratory’s nonconformities pre- to post-accreditation are illustrated in Figures S1, S2, S3, S4, S5, and S6 (see Additional file 1).

## Discussion

Laboratory accreditation of NMRA Quality Control laboratories improves their compliance to international standards which enhances mutual recognition of test reports. Regulatory decisions made by NMRAs within sub-Saharan Africa depend on reliability of laboratory data to confirm the quality of medicines available for healthcare and patient outcomes. Accuracy of test reports are improved through laboratory accreditation. This forms an important strategy for sub-Saharan African countries to collaborate and harmonize their regulatory efforts and avoid duplicating laboratory testing conducted in other accredited NMRA laboratories within the region. Results reported here affirm that laboratory accreditation improves the quality management system, consistent with findings from Nicklin et al., where benefits of accreditation included an improvement in the quality of health care services [46].

With respect to the first research question—does accreditation improve adherence to quality standards? —the significant decrease in the number of nonconformities in the post-accreditation years suggests that accreditation of the Quality Control laboratory improved adherence to a quality standard (ISO/IEC 17025:2005). Examination of the second research query—does accreditation decrease the severity of nonconformities? —the Chi-squared contingency evaluation between audit years, showed a significant decrease in severity of nonconformities in the post- accreditation years.

Attaining a laboratory accreditation comes with several costs, including facility upgrades, procurement of new equipment, calibration and maintenance schedules, as well as building capacity for personnel. These costs often form barriers for lower-resource settings to attain international standard requirements. The NMRA in the current study received support from the USP PQM program [31], sponsored by the United States Agency for International Development (USAID). An initial audit was conducted by USP PQM in 2013, after which the NMRA laboratory reviewed in this study received several trainings from the USP team to build personnel capacity in technical requirements of the ISO/IEC 17025:2005 standard. The leadership commitment to quality systems enabled the laboratory to satisfy the clauses for management requirements in the document. Leadership provided funding and resources to support the accreditation process, including procurement of equipment with maintenance and calibration plans and upgrades on both utilities and the facility as well as maintenance contracts. Similar costs and resources were needed to achieve accreditation in other institutions, including a 2018 survey by the Association Of Public Health Laboratories [47]. Other studies also enumerated comparable costs associated with accreditation process [47–51].

The NAFDAC laboratory faced other hurdles common to the region, such as political and cultural challenges. Tene and colleagues (2018) highlighted similar barriers faced by African countries in adopting ISO standards. These bulwarks include weak institutional frameworks, poorly managed-donor-funding, and low technical capacities [25]. Despite such challenges, the Nigerian NMRA laboratory from this study was able to attain ISO/IEC 17025 certification in 2016.

Although the observed increase in the number and severity of nonconformities from 2017 to 2018 were not significant, it was expected that these indices should have continually declined, in contrast to the audit reports. This will be an area of focus for further research for subsequent years’ audit trends.

Overall, the accreditation process improved the laboratory’s performance as evidenced by a significant decrease in the number of nonconformities from 2013 to later years 2017 and 2018. Furthermore, there was a reduction in the severity of nonconformities after laboratory accreditation. In summary, the laboratory quality management systems improved after ISO/IEC 17025 accreditation, consistent with previous research conducted in other types of laboratories [3, 24, 43, 52].

### Limitations

Admittedly, a lower sampling error would have occurred with a larger sample size. In addition, a random sampling would have been performed if the researchers had more options of publicly available data from other NMRAs Quality Control laboratory audits. At the time of this study, though this NMRA laboratory had undergone several audits from various inspection bodies, they were audited only thrice with the ISO/IEC 17025:2005 standard. Since there are variations in the requirements of different laboratory standards, this study assessed only the audit reports related to the ISO/IEC 17025:2005 standard. The three audit reports reviewed, however, covered all clauses under this standard against which the laboratory’s performances were measured.

## Conclusion

The present study aimed to investigate the impact of ISO/IEC 17025 accreditation on the quality management systems of an NMRA quality control laboratory as a means of improving the reliability of analytical reports emanating from testing activities of such labs. The study provided some evidence of improved quality compliance from a laboratory in a lower-resource setting after attaining ISO/IEC 17025:2005 accreditation. Therefore, the accreditation process improved the laboratory performance.

Also, laboratory accreditation requires commitment from upper level management to fulfill most of the requirements in the ISO/IEC 17025 standard. Together with other technical components, the major and minor nonconformities in a laboratory testing environment can be significantly reduced.

The following recommendations are provided for future consideration. Mutual recognition of laboratory Quality Control reports within NMRAs in sub-Saharan Africa would enhance harmonization buy-in within the region and positively impact the cost and timeliness of medicines regulation. Common recognition of analytical reports should be fostered among member countries which attain international accreditation for their NMRA Quality Control laboratories.

Collaboration within the sub-region may have produced more robust results if more countries’ NMRA Quality Control laboratories released their results (under confidentiality agreements) for pre- and post -accreditation evaluation of the ISO/IEC 17025 audits.

## Supplementary Information

**Additional file 1: Table S1.** Management Requirements 2013 Audit Summary. Evaluation of Quality Management System Based on ISO/IEC 17025 Standard. Date (s) of Inspection / Audit- June 17-22, 2013. **Table S2.** Technical Requirements 2013 Audit Summary. Evaluation of Quality Management System Based on ISO/IEC 17025 Standard. Date (s) of Inspection / Audit- June 17-22, 2013. **Table S3.** Summary for Evaluation of Quality Management System Based on ISO/IEC 17025 Standard 2013. **Table S4.** ISO/IEC 17025 Surveillance Audit - Date (s) of Inspection / Audit- December 14-15, 2017. **Table S5.** Summary for ISO/IEC 17025 Surveillance Audit 2017. **Table S6.** ISO/IEC 17025 Mock Audit - Date (s) of Inspection / Audit- September 24-26, 2018. **Table S7.** Summary for ISO/IEC 17025 Mock Audit (24-26 September 2018). **Table S8.** Management Requirements Nonconformities (NCs) (Pre - Post Accreditation). **Table S9.** Technical Requirements Nonconformities (Pre - Post Accreditation). **Table S10.** Types of Management Requirements Nonconformities (Major, Minor, Opportunities for Improvement (OFI)) Pre-Post Accreditation. **Table S11.** Types of Technical Requirements Nonconformities (Major, Minor, Opportunities for Improvement (OFI)) Pre-Post Accreditation. **Table S12.** Summary of the Severity of Types of Nonconformities (Major, Minor, Opportunities for Improvement (OFI)) Pre- and Post-Accreditation. **Table S13.** Summary of Severity of Nonconformities (NCs) Pre-Post Accreditation. **Figure S1.** Management Requirement Nonconformities Decreased Pre - Post Accreditation. **Figure S2.** Decline in Technical Requirement Nonconformities Pre - Post Accreditation. **Figure S3.** Management Requirement Nonconformities (NCs) Decreased Pre - Post Accreditation. **Figure S4.** Decrease in the Types of Management Requirement Nonconformities (Major, Minor, Opportunities for Improvement (OFI)) Pre-Post Accreditation. **Figure S5.** Decrease in Technical Requirement Nonconformities (Major, Minor, and Opportunities for Improvement (OFI)) Pre-Post Accreditation. **Figure S6.** Impact of Accreditation on Severity of Nonconformities (NCs) Illustrates Decline Pre-Post Accreditation Years.

## Data Availability

The datasets used and/or analyzed during the current study are available from the corresponding author on reasonable request. Reports for ISO/IEC 17025:2005 Audits, conducted by ANAB are available and can be provided on request.

## Abbreviations

#: Number
ANAB: ANSI National Accreditation Board
ANSI: American National Standards Institute
ILAC: International Laboratory Accreditation Cooperation
ISO / IEC: International Organization for Standardization / International Electrotechnical Commission
NAFDAC: National Agency for Food and Drugs Administration and Control
NCs: Nonconformities
NMRA: National Medicines Regulatory Authority
OFI: Opportunity for Improvement
PQM: Promoting the Quality of Medicines
USAID: United States Agency for International Development
USP: United States Pharmacopoeia
WHO: World Health Organization
