# Supplementary material for: Impact of ISO/IEC 17025 laboratory accreditation in sub-Saharan Africa: a case study
Source: BMC Health Serv Res. 2020 Nov 23;20:1065. doi: 10.1186/s12913-020-05934-8 (PMC7686690; doi:10.1186/s12913-020-05934-8)
Supplement: Supplementary file 1 — Additional file 1: Table S1. Management Requirements 2013 Audit Summary. Evaluation of Quality Management System Based on ISO/IEC 17025 Standard. Date (s) of Inspection / Audit- June 17-22, 2013. Table S2. Technical Requirements 2013 Audit Summary. Evaluation of Quality Management System Based on ISO/IEC 17025 Standard. Date (s) of Inspection / Audit- June 17-22, 2013. Table S3. Summary for Evaluation of Quality Management System Based on ISO/IEC 17025 Standard 2013. Table S4. ISO/IEC 17025 Surveillance Audit - Date (s) of Inspection / Audit- December 14-15, 2017. Table S5. Summary for ISO/IEC 17025 Surveillance Audit 2017. Table S6. ISO/IEC 17025 Mock Audit - Date (s) of Inspection / Audit- September 24-26, 2018. Table S7. Summary for ISO/IEC 17025 Mock Audit (24-26 September 2018). Table S8. Management Requirements Nonconformities (NCs) (Pre - Post Accreditation). Table S9. Technical Requirements Nonconformities (Pre - Post Accreditation). Table S10. Types of Management Requirements Nonconformities (Major, Minor, Opportunities for Improvement (OFI)) Pre-Post Accreditation. Table S11. Types of Technical Requirements Nonconformities (Major, Minor, Opportunities for Improvement (OFI)) Pre-Post Accreditation. Table S12. Summary of the Severity of Types of Nonconformities (Major, Minor, Opportunities for Improvement (OFI)) Pre- and Post-Accreditation. Table S13. Summary of Severity of Nonconformities (NCs) Pre-Post Accreditation. Figure S1. Management Requirement Nonconformities Decreased Pre - Post Accreditation. Figure S2. Decline in Technical Requirement Nonconformities Pre - Post Accreditation. Figure S3. Management Requirement Nonconformities (NCs) Decreased Pre - Post Accreditation. Figure S4. Decrease in the Types of Management Requirement Nonconformities (Major, Minor, Opportunities for Improvement (OFI)) Pre-Post Accreditation. Figure S5. Decrease in Technical Requirement Nonconformities (Major, Minor, and Opportunities for Improvement (OFI)) Pre-Post Accredit [file 12913_2020_5934_MOESM1_ESM.docx]

**Additional File 1**

| Table S1 Management Requirements 2013 Audit Summary | | | |
| --- | --- | --- | --- |
| ISO/IEC 17025:2005 Clause # | specific subdivision # | # of nonconformities / observations | Total observations / Clause |
| 4.0 (Management Requirements) | 4.1.3, 4.1.4, 4.1.5(ac), 4.1.5 (i), 4.1.5 (j), 4.1.6, | 6 | 46 |
|  |  |  |  |
|  | 4.2.1-7 | 1 |  |
|  | 4.3.1, 4.3.2.1, 4.3.2.2 (a-d) 4.3.3.3, 4.3.3.4 | 5 |  |
|  | 4.4.1(b, c), 4.4.2, 4.5, 4.5.3, 4.5.4, 4.4.1 | 6 |  |
|  | 4.6.2, 4.6.4 | 2 |  |
|  | 4.7.1, 4.7.2 | 2 |  |
|  | 4.8 | 1 |  |
|  | 4.9, 4.9.1(c) | 2 |  |
|  | 4.10 | 1 |  |
|  | 4.11, 4.11.4, 4.11.3, 4.11.5, 4.11.2 | 5 |  |
|  |  |  |  |
|  | 4.12.1, 4.12.2 | 2 |  |
|  | 4.13.1.4, 4.13.2.3, 4.13.1.1, 4.13.1.2, 4.13.1.3, 4.13.2.1, 4.13.2.2 | 7 |  |
|  | 4.14.1, 4.14.2, 4.14.3, 4.14.4 | 4 |  |
|  | 4.15.1, 4.15.2 | 2 |  |

Evaluation of Quality Management System Based on ISO/IEC 17025 Standard. Date (s) of Inspection / Audit- June 17-22, 2013

| Table S2 Technical Requirements 2013 Audit Summary | | | |
| --- | --- | --- | --- |
| ISO/IEC 17025:2005 Clause # | specific subdivision # | # of nonconformities / observations | Total observations / Clause |
| 5.0 (Technical Requirements | 5.2.1, 5.2.2, 5.2.3, 5.2.5 | 4 | 47 |
|  | 5.3.2, 5.3.3, 5.3.4 | 3 |  |
|  | 5.4.7.1 5.4.7.2 (a-c) | 7 |  |
|  | 5.4.1 5.4.2 5.4.4 |  |  |
|  | 5.4.6.2 5.4.6.3 |  |  |
|  | 5.5.2, 5.5.3, 5.5.9, 5.5.5, 5.5.6, 5.5.8, 5.5.10, 5.5.11 | 8 |  |
|  | 5.6.1, 5.6.2, 5.6.2.2.1, 5.6.2.2 5.6.2.2.2 | 8 |  |
|  | 5.6.3.1, 5.6.3.3 5.6.3.4 |  |  |
|  | 5.7.1, 5.7.3 | 2 |  |
|  | 5.8.2, 5.8.3, 5.8.4 | 3 |  |
|  | 5.9.1, 5.9.2, 5.9.1 (ae) | 3 |  |
|  | 5.10.2, 5.10.3.1, 5.10.3.2, 5.10.4.1, 5.10.4.2, 5.10.4.3, 5.10.4.4, 5.10.7, 5.10.8, | 9 |  |

Evaluation of Quality Management System Based on ISO/IEC 17025 Standard. Date (s) of Inspection / Audit- June 17-22, 2013

Table S3 Summary for Evaluation of Quality Management System Based on ISO/IEC 17025 Standard 2013

| ISO/IEC 17025:2005 Clause # | Area of Evaluation | Major (#) | Minor (#) | OFI (#) |
| --- | --- | --- | --- | --- |
| 4 | Management Requirements | 38 | 8 | 0 |
| 5 | Technical Requirements | 25 | 22 | 0 |

Table S4 ISO/IEC 17025 Surveillance Audit - Date (s) of Inspection / Audit- December 14-15, 2017.

| ISO/IEC 17025:2005 Clause # | specific subdivision # | # of nonconformities / observations | Total observations / Clause |
| --- | --- | --- | --- |
| 4.0  (Management Requirements) | 4.3.2.1  4.13.1.2  4.15.2 | 1  1  1 | 3 |
| 5.0  (Technical Requirements) | 5.3.2  5.4.1  5.5.5c | 1  2  1 | 4 |

Table S5 Summary for ISO/IEC 17025 Surveillance Audit 2017

| ISO/IEC 17025:2005 Clause # | Area of Evaluation | Major (#) | Minor (#) |
| --- | --- | --- | --- |
| 4 | Management Requirements | 2 | 1 |
| 5 | Technical Requirements | 2 | 2 |

Table S6 ISO/IEC 17025 Mock Audit - Date (s) of Inspection / Audit- September 24-26, 2018

| ISO/IEC 17025:2005 Clause # | specific subdivision # | # of nonconformities / observations | Total observations / Clause |
| --- | --- | --- | --- |
| 4.0  (Management Requirements) | 4.1.4.1  4.1.5.1  4.1.5 (b)  4.8.1.1  4.9.1  4.13.1.2 | 1  1  1  1  1  1 | 6 |
| 5.0  (Technical Requirements) | 5.2.1  5.3.1  5.3.6.1  5.4.2  5.5.5(f)  5.9.1  5.9.1 (b) | 1  1  1  1  1  2  1 | 8 |

Table S7 Summary for ISO/IEC 17025 Mock Audit (24-26 September 2018)

| ISO/IEC 17025:2005 Clause # | Area of Evaluation | Major (#) | Minor (#) | OFI (#) |
| --- | --- | --- | --- | --- |
| 4 | Management Requirements | 3 | 3 | 0 |
| 5 | Technical Requirements | 6 | 2 | 0 |

Table S8 Management Requirements Nonconformities (NCs) (Pre - Post Accreditation)

| ISO/IEC 17025 CLAUSE | 2013 | 2017 | 2018 |
| --- | --- | --- | --- |
| 4.1 - < 4.2 | 6 | 0 | 3 |
| 4.2 - < 4.3 | 1 | 0 | 0 |
| 4.3 - < 4.4 | 5 | 1 | 0 |
| 4.4 - < 4.5 | 6 | 0 | 0 |
| 4.5 - < 4.6 | 0 | 0 | 0 |
| 4.6 - < 4.7 | 2 | 0 | 0 |
| 4.7 - < 4.8 | 2 | 0 | 0 |
| 4.8 - < 4.9 | 1 | 0 | 1 |
| 4.9 - < 4.10 | 2 | 0 | 1 |
| 4.10 - < 4.11 | 1 | 0 | 0 |
| 4.11 - < 4.12 | 5 | 0 | 0 |
| 4.12 - < 4.13 | 2 | 0 | 0 |
| 4.13 - < 4.14 | 7 | 1 | 1 |
| 4.14 - < 4.15 | 4 | 0 | 0 |
| 4.15 - < 4.16 | 2 | 1 | 0 |

Table S9 Technical Requirements Nonconformities (Pre - Post Accreditation)

| ISO/IEC 17025 CLAUSE | 2013 | 2017 | 2018 |
| --- | --- | --- | --- |
| 5.1 - < 5.2  5.2 - < 5.3  5.3 - < 5.4  5.4 - < 5.5  5.5 - < 5.6  5.6 - < 5.7  5.7 - < 5.8  5.8 - < 5.9  5.9 - < 5.10  5.10 - < 5.11 | 0  4  3  7  8  8  2  3  3  9 | 0  0  1  2  1  0  0  0  0  0 | 0  1  2  1  1  0  0  0  3  0 |

Table S10 Types of Management Requirements Nonconformities (Major, Minor, Opportunities for Improvement (OFI)) Pre-Post Accreditation

| Nonconformities (type) | 2013 | 2017 | 2018 |
| --- | --- | --- | --- |
| Major | 38 | 2 | 3 |
| Minor | 8 | 1 | 3 |
| OFI | 0 | 0 | 0 |

Table S11 Types of Technical Requirements Nonconformities (Major, Minor, Opportunities for Improvement (OFI)) Pre-Post Accreditation

| Nonconformities (type) | 2013 | 2017 | 2018 |
| --- | --- | --- | --- |
| Major | 25 | 2 | 6 |
| Minor | 22 | 2 | 2 |
| OFI | 0 | 0 | 0 |

Table S12 Summary of the Severity of Types of Nonconformities (Major, Minor, Opportunities for Improvement (OFI)) Pre- and Post-Accreditation

| Type of NC | 2013 | 2017 | 2018 |
| --- | --- | --- | --- |
| mgt. major NCs | 38 | 2 | 3 |
| tech. major NCs | 25 | 2 | 6 |

Figure S1

Management Requirement Nonconformities Decreased Pre - Post Accreditation

Figure S2

Decline in Technical Requirement Nonconformities Pre - Post Accreditation

Figure S3

Management Requirement Nonconformities (NCs) Decreased Pre - Post Accreditation

Figure S4

Decrease in the Types of Management Requirement Nonconformities (Major, Minor, Opportunities for Improvement (OFI)) Pre-Post Accreditation

Figure S5

Decrease in Technical Requirement Nonconformities (Major, Minor, and Opportunities for Improvement (OFI)) Pre-Post Accreditation

Table S13 Summary of Severity of Nonconformities (NCs) Pre-Post Accreditation

|  | Management Requirements NCs (#) | | Technical Requirements  NCs (#) | |
| --- | --- | --- | --- | --- |
| Year of audit | Major | Minor | Major | Minor |
| 2013 | 38 | 8 | 25 | 22 |
| 2017 | 2 | 1 | 2 | 2 |
| 2018 | 3 | 3 | 6 | 2 |

Figure S6

Impact of Accreditation on Severity of Nonconformities (NCs) Illustrates Decline Pre-Post Accreditation Years
